# Assessing the toxicological effects of exposure to polyethylene terephthalate on hepatocellular carcinoma: insights from network toxicology, molecular docking, molecular dynamics, and experimental validation

**DOI:** 10.3389/fphar.2026.1772751

**Published:** 2026-04-22

**Authors:** Peng Chen, Xin Liu, Dongmei Yuan, Lan Zheng, Yumin Du, Dacai Gong, Xing Wei, Dan Wang, Ge Xu, Bin Ge

**Affiliations:** 1 Department of Clinical Laboratory, Pidu District People’s Hospital, The 3RD Affiliated Hospital of Chengdu Medical College, Chengdu, Sichuan, China; 2 Department of Clinical Laboratory, 363 Hospital, Chengdu, Sichuan, China; 3 Department of Pathogenic Biology, School of Basic Medical Sciences, Chengdu Medical College, Chengdu, China

**Keywords:** CCNA2, Cdc25C, hepatocellular carcinoma, molecular docking, plk1, polyethylene terephthalate

## Abstract

**Background:**

Polyethylene terephthalate (PET), one of the most widely used synthetic polymers globally, has emerged as a potential environmental risk factor for human health. However, the molecular mechanisms linking PET exposure to hepatocellular carcinoma (HCC) remain poorly understood.

**Methods:**

We adopted an integrated systems biology framework that combined computational target prediction (using ChEMBL, PharmMapper, and SwissTargetPrediction), transcriptomic profiling, and machine learning to elucidate key molecular targets and pathways involved in PET-associated hepatocarcinogenesis. Immune cell infiltration was assessed via CIBERSORT. Molecular docking followed by 100 ns molecular dynamics simulations were employed to verify protein-ligand binding interactions. The expression profiles and prognostic relevance of core genes were evaluated using TCGA-LIHC datasets. *In vitro* validation was carried out in two HCC cell lines (Hep3B and HepG2) through qRT-PCR, Western blotting, EdU incorporation assays, colony formation assays, and flow cytometric cell cycle analysis.

**Results:**

We identified 235 potential PET-interacting proteins, with 40 genes overlapping with HCC-associated genes. Integrated analysis consistently identified PLK1, CCNA2, and CDC25C as core mediators of PET-associated hepatocarcinogenesis. Molecular docking revealed potential binding interactions, with PLK1 showing the highest affinity (--8.0 kcal/mol). Molecular dynamics simulations confirmed sustained structural stability of these PET-protein complexes over 100 ns. Clinical data analysis demonstrated progressive upregulation of these genes with advancing tumor stage and grade, with high expression predicting poor overall survival. PET treatment of Hep3B and HepG2 cells significantly upregulated PLK1, CCNA2, and CDC25C expression at both mRNA and protein levels, enhanced colony formation capacity, increased EdU-positive cells, and promoted G2/M phase progression. Western blotting further revealed upregulation of the proliferation marker PCNA. Functional enrichment analysis revealed involvement of cell cycle regulation, metabolic reprogramming, and immune microenvironment remodeling. CIBERSORT analysis identified significant correlations between core gene expression and infiltration of neutrophils, monocytes, and macrophages, alongside negative associations with lymphoid populations.

**Conclusion:**

PET exposure may promote hepatocarcinogenesis through multi-layered mechanisms involving cell cycle dysregulation (primarily via PLK1, CCNA2, and CDC25C), metabolic reprogramming, and immune microenvironment remodeling. These findings provide mechanistic insights into plastic-associated cancer risk and identify potential biomarkers and therapeutic targets for populations with PET exposure.

## 1 Introduction

Polyethylene terephthalate (PET) is one of the most extensively produced synthetic polymers worldwide, widely used in packaging materials, textiles, and medical devices due to its durability, lightweight nature, and chemical resistance. However, increasing evidence has demonstrated that PET and its degradation products, especially micro- and nanoplastics, can enter biological systems and accumulate in various organs through ingestion, inhalation, or dermal contact (Domenech and Marcos, 2021; Kumar et al., 2022; Domenech et al., 2023; Ramsperger et al., 2023). Recent studies have detected PET microplastics in human blood, feces, and liver tissues, raising growing concern about their potential health implications (Leslie et al., 2022; Kutralam-Muniasamy et al., 2023). Despite its chemical stability under standard conditions, PET can undergo hydrolysis, photodegradation, and oxidative fragmentation, generating smaller particles and leachable additives that may interfere with cellular metabolism and molecular signaling (Schwabl et al., 2019).

The liver plays a pivotal role in xenobiotic metabolism and detoxification, making it particularly vulnerable to environmental pollutants, including microplastics. Accumulating evidence suggests that exposure to plastic-derived particles may induce oxidative stress, inflammation, and lipid peroxidation in hepatic tissues (Lu et al., 2016; Barboza et al., 2018; Ding et al., 2018; Brun et al., 2019; Xia et al., 2020; Yang et al., 2020). These effects have been associated with mitochondrial dysfunction and DNA damage, which are known to promote tumorigenic processes (Shen et al., 2022). However, the specific molecular mechanisms linking PET exposure to hepatocellular carcinoma (HCC)—the most prevalent and lethal form of primary liver cancer—remain largely unexplored. Traditional toxicological studies have primarily focused on histopathological alterations or biochemical parameters, lacking systematic elucidation of PET’s molecular targets and their downstream signaling pathways.

Deciphering the complex interaction between environmental toxins and cancer biology requires a systemic approach. Traditional toxicological screenings are often time-consuming and limited in their ability to map complex molecular networks. In contrast, the integration of network pharmacology, multi-omics bioinformatics, and machine learning (ML) offers a powerful paradigm to predict potential toxicological targets and disease associations with high precision. By constructing “pollutant-target-disease” networks, researchers can identify key signaling cascades and hub genes that serve as bridges between environmental exposure and disease phenotypes. Furthermore, molecular dynamics (MD) simulations provide a virtual microscope to inspect the thermodynamic stability of these pollutant-protein interactions at the atomic level (Filipe and Loura, 2022; Wu et al., 2022).

In the present study, we employed a comprehensive, multi-disciplinary strategy to elucidate the molecular mechanisms linking PET exposure to HCC progression. We first identified potential protein targets of PET and intersected them with HCC-related transcriptomic data from The Cancer Genome Atlas (TCGA). Subsequently, we utilized Weighted Gene Co-expression Network Analysis (WGCNA) and a robust ensemble of machine learning algorithms to screen for core diagnostic biomarkers. We further investigated the impact of these core genes on the tumor immune microenvironment (TIME) and validated the direct binding affinity of PET to key oncogenic targets via molecular docking and MD simulations. Finally, the functional relevance of these computational findings was verified *in vitro* using Hep3B cells, providing direct biological evidence that PET exposure activates specific cell-cycle regulatory pathways to promote hepatocarcinogenesis.

## 2 Materials and methods

### 2.1 Acquisition of PET targets

The molecular structure of PET and Simplified Molecular Input Line Entry System (SMILES) “CC(—O)C1—CC—C(C—C1)C (—O)OCCOC” were obtained from the PubChem database (Kim et al., 2025). Potential human protein targets were predicted with three complementary approaches to maximize recall and reduce method-specific bias: (1) ChEMBL (ligand–target bioactivity inference), (2) PharmMapper (reverse pharmacophore mapping), and (3) SwissTargetPrediction (2D/3D similarity and statistical learning) (Daina and Zoete, 2024). Predicted targets were merged by UniProt gene symbols, deduplicated, and restricted to *Homo sapiens* entries.

### 2.2 Differential gene expression analysis

Gene expression profiles and clinical data for Hepatocellular Carcinoma (HCC) were obtained from The Cancer Genome Atlas (TCGA) database, comprising HCC tumor tissues and adjacent normal liver tissues. Based on the transcriptomic data analyzed with the limma package, we identified differentially expressed genes (DEGs) using the following screening criteria: FDR-adjusted P < 0.05 and |log2FC| > 0.585 (1.5-fold change) (Ritchie et al., 2015). The resulting data were then visualized via ggplot2.

### 2.3 Weighted gene co-expression network analysis

A scale-free co-expression network was built with the WGCNA package, which involved: hierarchical clustering for outlier removal; dynamic tree-cutting for optimal soft-power selection (*R* ^2^ > 0.85); module detection via TOM-based hierarchical clustering (minModuleSize = 30, mergeCutHeight = 0.25); module-trait correlation analysis (|R| > 0.5, P < 0.05); and hub gene identification via intramodular connectivity (kME >0.8).

### 2.4 Functional enrichment and PPI network construction

The intersection of PET-associated targets and HCC-related genes (from DEGs and WGCNA modules) was identified using Venn diagrams. To elucidate the biological functions of these overlapping genes, Gene Ontology (GO) annotation and Kyoto Encyclopedia of Genes and Genomes (KEGG) pathway enrichment analyses were performed using the “clusterProfiler” package in R (Ashburner et al., 2000; Kanehisa et al., 2025). A Protein-Protein Interaction (PPI) network was constructed using the STRING database with a minimum interaction score of 0.400. The network was visualized using Cytoscape software. The MCODE plugin was utilized to identify significant functional clusters. Seven topological centrality algorithms (closeness, degree, eigenvector, EPC, MCC, MNC, and bottleneck) were applied via the CytoNCA plugin to identify potential hub genes.

### 2.5 Machine learning-based biomarker screening

To identify PET-associated diagnostic markers for HCC, we established a comprehensive machine learning framework integrating multiple algorithms. First, using the training set expression profiles, we constructed 113 prediction models with 10 classical algorithms and performed hyperparameter optimization via 5-fold cross-validation with stratified sampling for internal validation. Subsequently, we comprehensively evaluated all models based on the area under the ROC curve (AUC), accuracy, and F1-score. Based on this evaluation, we adopted a stacking ensemble learning strategy to integrate predictions from the top-performing single models, thereby enhancing predictive robustness. Finally, we selected high-confidence models (AUC >0.9), identified candidate core genes by ranking the frequency of their feature genes, and visualized the expression patterns of these candidates in a heatmap using the pheatmap package.

### 2.6 Immune infiltration analysis

To estimate immune composition, bulk RNA-seq profiles were deconvolved with CIBERSORT using the LM22 signature matrix (22 immune cell types). Only samples with CIBERSORT deconvolution p < 0.05 were retained. Relative fractions were compared between groups, and associations between core gene expression and immune fractions were evaluated using Spearman correlation with FDR control.

### 2.7 Molecular docking and molecular dynamics simulation

The PET monomer structure (PubChem CID 18721140), representing the smallest repeating chemical unit of the polymer, was used as the ligand. This approach is consistent with prior computational toxicology studies modeling plastic-derived chemicals, as environmental degradation processes (hydrolysis, photodegradation, mechanical fragmentation) generate monomers and oligomers that may be bioavailable at the molecular level. Docking was performed using AutoDock Vina with grid boxes centered on known/putative ligand-binding pockets (defined by co-crystallized ligands or pocket detection). Exhaustiveness settings were chosen to balance runtime and search completeness. Binding free energies (kcal/mol) were recorded for top-ranked poses; values ≤ −5.0 kcal/mol were interpreted as indicative of favorable binding. Docked complexes were inspected visually in PyMOL to assess pose plausibility and intermolecular interactions.GROMACS 2022 was used for 100 ns molecular dynamics simulations under standard temperature and pressure using Charmm 36 (Jo et al., 2008) and TIP3P models.

### 2.8 Cell culture

The human hepatocellular carcinoma cell line Hep3B was purchased from the American Type Culture Collection (ATCC, Manassas, VA, USA, catalog number: HB-8064). Hep3B cells were cultured in high glucose Dulbecco’s Modified Eagle Medium (DMEM, Gibco) supplemented with 10% fetal bovine serum (FBS, Gibco) and 1% penicillin-streptomycin solution (Gibco). All cells were maintained in a humidified incubator at 37 °C with 5% CO_2_. Culture medium was replaced every 2–3 days. When cells reached 80%–90% confluence, they were passaged at a 1:3 ratio following digestion with 0.25% trypsin-EDTA solution (Gibco). All experiments were performed using cells within six passages. Cell morphology was regularly monitored by microscopy, and *mycoplasma* testing was conducted to ensure cells were in good condition and free from contamination.

### 2.9 PET particle preparation

PET microplastics were purchased from Rigor Biotechnology Co. Ltd. (Wuxi, China). Briefly, recycled PET plastic bottles were thoroughly cleaned and dried, then sheared into small flakes using a cutting machine. Subsequently, PET flakes were further ground into particles using a cryogenic freezing and grinding method. The resulting coarse particles were subjected to sieving and graded using standard sieves to obtain particles of uniform size. The final PET particles were dried and stored in a desiccator for subsequent experiments. The entire preparation process was conducted in a dust-free environment to prevent contamination by impurities.

### 2.10 PET particle treatment

To assess the effects of PET particles on Hep3B cells, pre-prepared and sieved PET particles were suspended in sterile phosphate-buffered saline (PBS) and dispersed by ultrasonication (power: 200 W, frequency: 40 kHz) for 30 min to prepare a stock suspension. The selected concentrations (10 μg/mL, 50 μg/mL, and 100 μg/mL) were chosen based on previously reported *in vitro* microplastic exposure studies, which commonly employ concentrations ranging from 1 to 100 μg/mL to assess subcytotoxic effects. According to experimental grouping requirements, the PET suspension was added to cell culture medium to achieve final concentrations of 10 μg/mL, 50 μg/mL, and 100 μg/mL, respectively. Prior to treatment, Hep3B cells were seeded in 6-well plates and cultured to appropriate density (approximately 70% confluence), then the medium was replaced with fresh medium containing different concentrations of PET particles for intervention treatment. All treatment groups, along with the particle-free control group, were maintained under identical conditions (37 °C, 5% CO_2_) and cultured for 24 h, or cells were collected at experimentally predetermined time points for subsequent analysis. To ensure homogeneous particle distribution, suspensions were briefly vortexed before each use. PET suspensions were freshly prepared and used within 4 h of preparation to ensure particle dispersion stability.

### 2.11 Colony formation assay

Hep3B cells were seeded into 6-well plates at a density of 500 cells per well. PET particles were then added at concentrations of 10 μg/mL, 50 μg/mL, and 100 μg/mL, while untreated cells served as controls. After 7 days of incubation, cells were fixed with 4% paraformaldehyde and stained with 0.1% crystal violet. Colonies containing ≥50 cells were counted under a light microscope. The number of colonies in the control group was set to 100%, and the colony formation rate in PET-treated groups was expressed as a percentage relative to the control.

### 2.12 RNA extraction and real-time quantitative PCR

Total RNA was extracted from Hep3B cells following PET particle treatment (10 μg/mL, 50 μg/mL, and 100 μg/mL for 24 h) using TRIzol reagent (Invitrogen, USA) according to the manufacturer’s instructions. RNA concentration and purity were assessed by NanoDrop spectrophotometry (Thermo Fisher Scientific, USA). First-strand cDNA was synthesized from 1 μg of total RNA using a PrimeScript RT reagent kit (Takara Bio, Japan). Real-time quantitative PCR (qRT-PCR) was performed using TB Green Premix Ex Taq II (Takara Bio) on a QuantStudio 5 Real-Time PCR System (Applied Biosystems, USA). The thermal cycling conditions were: 95 °C for 30 s, followed by 40 cycles of 95 °C for 5 s and 60 °C for 34 s. Target gene expression levels were normalized to GAPDH using the 2^(-ΔΔCt)^ method. Primer sequences are listed in Supplementary Table S1. All experiments were performed in triplicate.

### 2.13 Western blot

Total cellular proteins were isolated using RIPA lysis buffer containing protease inhibitor cocktail (0 469 313 2001, Roche). Protein concentrations were quantified using a BCA assay kit (23 227, Thermo, USA). Equal amounts of protein were resolved by SDS-PAGE and electroblotted onto PVDF membranes (Merck Millipore, USA). Membranes were blocked with 5% skim milk in TBST for 2 h at room temperature to eliminate nonspecific binding, then probed with primary antibodies (rabbit anti-PCNA (ET1605-38; HUABIO), rabbit anti-CCNA2 (ET1612-26; HUABIO), rabbit anti-CDC25C (JE59-53, HUABIO), anti-PLK1 (PSH04-35, HUABIO), and mouse anti-GAPDH (5-E10, HUABIO))overnight at 4 °C. Following three washes with TBST, membranes were incubated with HRP-conjugated secondary antibodies specific to the primary antibody species, and signals were visualized using ECL reagent (WBKLS0500, Millipore, Massachusetts, USA).

### 2.14 EdU detection

HCC cells post-transfection were seeded into 24-well plates at 2 × 10^5^ cells per well for EdU incorporation assays using the BeyoClick™ EdU Cell Proliferation Kit with DAB (C0085S, Beyotime). The experimental procedure encompassed EdU pulse-labeling, paraformaldehyde fixation, click-reaction staining, and DAPI nuclear counterstaining, with subsequent visualization via inverted fluorescence microscopy. Fluorescent images were analyzed and quantified using ImageJ software. Each experimental condition was set up in triplicate to ensure statistical reliability.

### 2.15 Statistical analysis

All statistical analyses and data visualizations were performed using R software and GraphPad Prism. Quantitative experimental data are presented as the mean ± standard deviation (SD) derived from at least three independent experiments. For comparisons between two groups, the Student’s t-test was employed. For comparisons among multiple groups, one-way Analysis of Variance (ANOVA) was performed, followed by Tukey’s *post hoc* test for pairwise comparisons. Correlation analyses between gene expression and immune infiltration were evaluated using Pearson or Spearman correlation coefficients. Survival analysis was conducted using the Kaplan-Meier method, with significance determined by the log-rank test. A p-value <0.05 was considered statistically significant.

## 3 Results

### 3.1 Identification of target proteins associated with PET exposure

**FIGURE 1:**
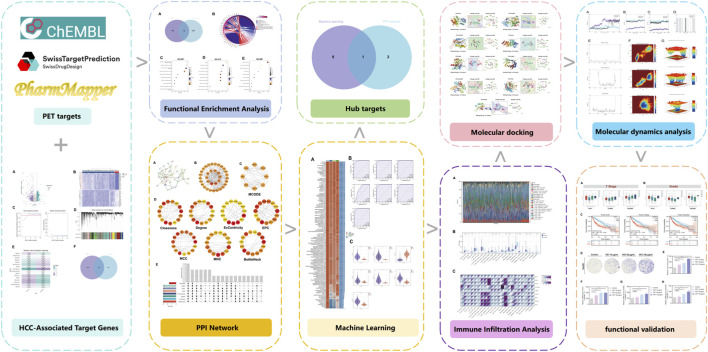
Flow-chart of dataset analysis in this paper.

**FIGURE 2:**
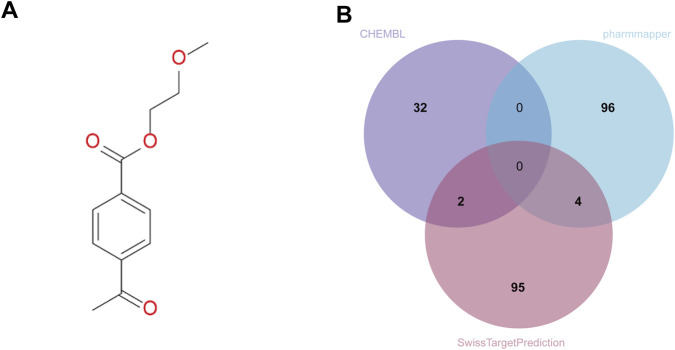
Structure of PET and Prediction of Potential Targets. **(A)** Chemical structure of PET obtained from the PubChem database. **(B)** Merged and de-duplicated target sets from ChEMBL, PharmMapper, and SwissTargetPrediction, identifying 235 unique candidate targets.

The detailed flowchart was showed in Figure 1. The molecular structure of polyethylene terephthalate (PET) was first retrieved from the PubChem database (Figure 2A). To explore its potential molecular targets, three complementary computational prediction platforms—ChEMBL, PharmMapper, and SwissTargetPrediction—were employed. After integrating the results from these databases and removing duplicate entries, a total of 235 distinct candidate target proteins potentially associated with PET exposure were identified (Figure 2B).This multi-database integration approach ensured high coverage and minimized false positives, providing a robust foundation for subsequent bioinformatic and functional analyses.

### 3.2 Identification of HCC-associated target genes

**FIGURE 3:**
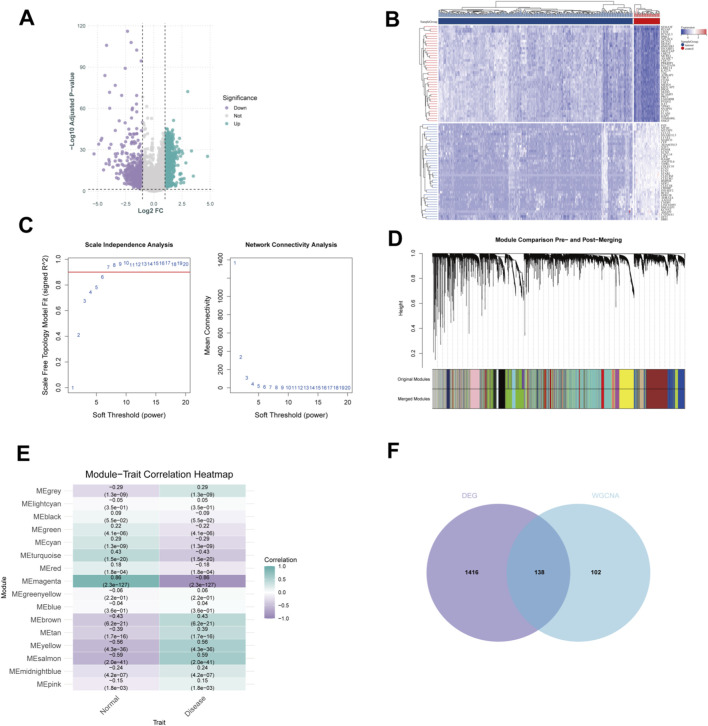
Differential Expression and WGCNA Analysis of HCC-Related Genes Based on TCGA. **(A)** Volcano plot showing 1,554 DEGs (1,075 upregulated in green; 479 downregulated in purple) with |logFC| > 1 and p.adj <0.05. **(B)** Hierarchical clustering heatmap of the top 40 DEGs. **(C)** Selection of the soft-thresholding power in WGCNA was guided by the scale-free topology model fit (signed *R* ^2^) and mean connectivity evaluated across powers 1–20. **(D)** Hierarchical clustering of the topological overlap matrix (TOM) showing module division. **(E)** Module–trait correlation heatmap (P < 0.05). **(F)** Intersection of DEGs and significant WGCNA modules, yielding 1,656 HCC-related genes.

To elucidate the molecular relationship between PET exposure and hepatocellular carcinoma (HCC), transcriptomic data were obtained from the TCGA database, encompassing both normal hepatic and HCC samples. Differential expression analysis revealed 1,554 differentially expressed genes (DEGs) (|LogFC| > 1, adjusted p < 0.05), comprising 1,075 upregulated and 479 downregulated genes (Figure 3A).Hierarchical clustering based on the top 40 DEGs demonstrated clear segregation between normal and HCC groups (Figure 3B), confirming the robustness of the transcriptomic distinction.To identify gene modules significantly correlated with HCC phenotypes, Weighted Gene Co-expression Network Analysis (WGCNA) was conducted. A soft-thresholding power of β = 6 was determined as optimal to achieve a scale-free topology (*R* ^2^ ≥ 0.8; Figure 3C). Subsequently, a Topological Overlap Matrix (TOM) was constructed, and hierarchical clustering delineated 16 co-expression modules (Figures 3D,E). Correlation analysis between module eigengenes and clinical traits highlighted several modules significantly associated with HCC (p < 0.05; Figure 3F). By intersecting DEGs with genes from HCC-associated WGCNA modules and removing duplicates, a refined set of 1,656 HCC-related genes was obtained for downstream integrative analyses.

### 3.3 Enrichment analysis of PET-associated disease targets in HCC

**FIGURE 4:**
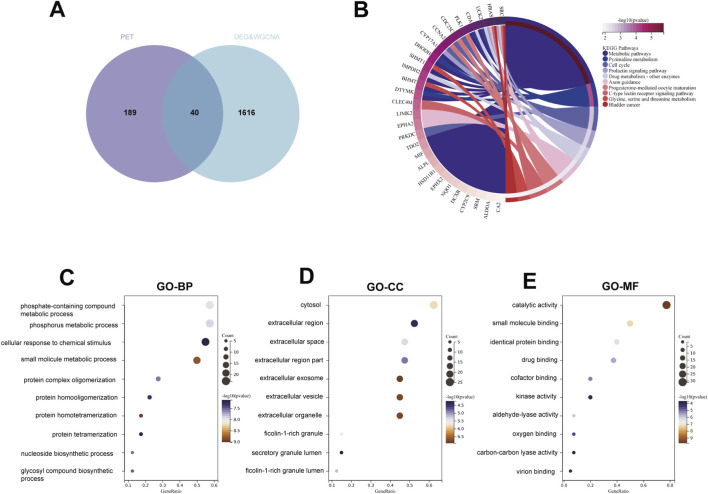
Overlap and Enrichment Analysis of PET and HCC Targets. **(A)** Venn diagram showing the overlap between PET-associated targets (merged from ChEMBL, PharmMapper, and SwissTargetPrediction) and HCC-associated genes (derived from the intersection of DEGs and WGCNA modules). A total of 40 shared targets were identified. **(B)** Chord diagram of the top enriched KEGG pathways for the 40 core genes. **(C–E)** Bubble chart of the top enriched GO terms.

To explore the biological implications of PET exposure in the context of hepatocellular carcinoma (HCC), an intersection analysis was performed between the predicted PET target proteins and the identified HCC-related genes. This comparison yielded 40 overlapping genes, representing potential key targets mediating PET-associated hepatocarcinogenesis (Figure 4A). Gene Ontology (GO) and Kyoto Encyclopedia of Genes and Genomes (KEGG) enrichment analyses were subsequently conducted to elucidate the molecular mechanisms underlying these targets (Figures 4B–E). GO enrichment revealed significant overrepresentation in categories including catalytic activity, phosphate-containing compound metabolic process, phosphorus metabolism, response to chemical stimuli, and small molecule metabolic process, with localization primarily in the cytosol and extracellular region.KEGG pathway analysis further indicated strong enrichment in Metabolic pathways, Pyrimidine metabolism, Cell cycle, Prolactin signaling pathway, Drug metabolism–other enzymes, Axon guidance, C-type lectin receptor signaling, and Glycine, serine, and threonine metabolism, among others. These results collectively suggest that PET may disrupt key metabolic and signaling processes related to hepatocarcinogenesis, potentially through alterations in energy metabolism, nucleotide synthesis, and cell-cycle regulation.

### 3.4 Screening of core target genes of PET-induced HCC through PPI network

**FIGURE 5:**
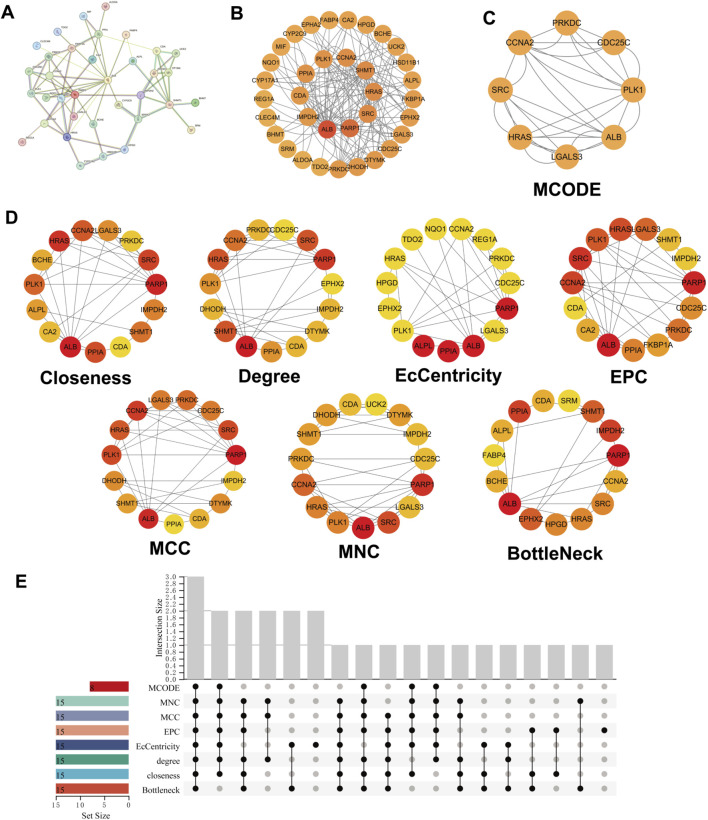
PPI Network and Hub Gene Screening among 40 Common Targets. **(A)** Medium-confidence PPI network constructed using the STRING database (interaction score ≥0.400). **(B)** Visualization of the resulting PPI network containing 35 nodes and 148 edges. **(C)** Key subnetwork module identified using the MCODE plugin in Cytoscape. **(D)** Top 15 core targets ranked by seven CytoHubba centrality algorithms, including Closeness, Degree, EcCentricity, EPC, MCC, MNC, and BottleNeck; darker colors indicate higher ranking. **(E)** UpSet plot showing the intersections of hub genes identified by the seven centrality measures.

To clarify the interactions among the 40 overlapping targets, a Protein–Protein Interaction (PPI) network was constructed using the STRING database with a medium confidence threshold (interaction score ≥0.400) (Figure 5A). The resulting network comprised 35 nodes and 148 edges, demonstrating a complex pattern of molecular interplay (Figure 5B). To further analyze the structural organization of the network, a key functional subnetwork module was identified using the MCODE plugin in Cytoscape, revealing a densely connected cluster of proteins with potential biological significance (Figure 5C). To determine the most central and influential nodes within the PPI network, seven complementary topological centrality measures—including closeness, degree, eigenvector, edge percolation centrality (EPC), maximum clique centrality (MCC), maximum neighborhood component (MNC), and bottleneck—were calculated (Figure 5D). These metrics collectively highlight nodes that play critical roles in maintaining network connectivity and functional integrity. An UpSet plot was subsequently employed to visualize the overlapping hub genes identified across these centrality algorithms, ultimately revealing ALB, HRAS, and CCNA2 as consistently ranked hub genes with high centrality values (Figure 5E).

### 3.5 Application of machine learning for the screening of core target genes in PET-induced HCC

To further identify the most critical genes involved in PET-associated hepatocarcinogenesis, a comprehensive machine learning–based screening was conducted using the 40 overlapping candidate targets obtained from prior analyses.

**FIGURE 6:**
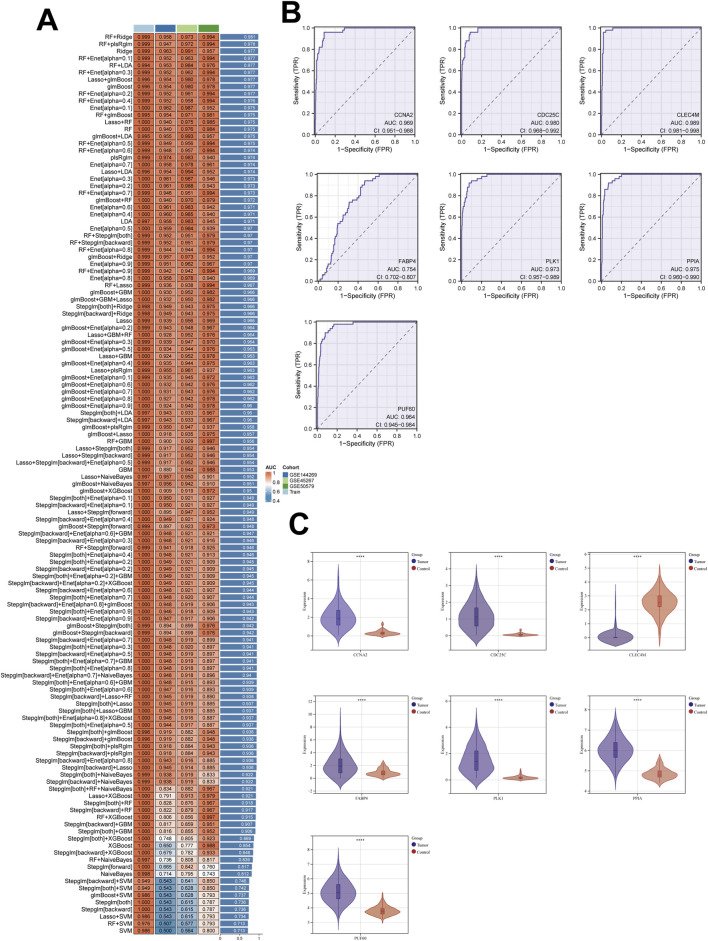
Machine Learning-Based Identification of Core Genes in PET-Induced HCC. **(A)** Performance comparison of 113 predictive models built with multiple algorithms. **(B)** Receiver Operating Characteristic (ROC) curves of the seven key genes. **(C)** Box plots illustrating the significant differential expression of the seven core genes between normal and HCC tissues.

A total of 113 predictive models were constructed employing multiple supervised learning algorithms, including Random Forest (RF), Support Vector Machine (SVM), Ridge regression, LASSO, and ensemble learning frameworks. Model performance was evaluated using fivefold cross-validation, with accuracy, AUC, and F1-score serving as the primary performance metrics (Figure 6A). Among all models, the Random Forest–Ridge regression ensemble model exhibited the best overall predictive performance in both training and validation datasets. Based on the feature importance and coefficient weighting derived from this optimized model, seven key genes—PUF60, CLEC4M, FABP4, CCNA2, PPIA, CDC25C, and PLK1 were identified as potential core targets associated with PET-induced HCC progression.The diagnostic reliability of these core genes was further verified by Receiver Operating Characteristic (ROC) curve analysis, which demonstrated high discriminatory power in distinguishing HCC from normal hepatic samples (Figure 6B). Additionally, box plots illustrated significant differential expression of all seven genes between normal and HCC tissues, reinforcing their potential roles as biomarkers in PET-related hepatocarcinogenesis (Figure 6C).

### 3.6 Functional enrichment analysis of core target genes

**FIGURE 7:**
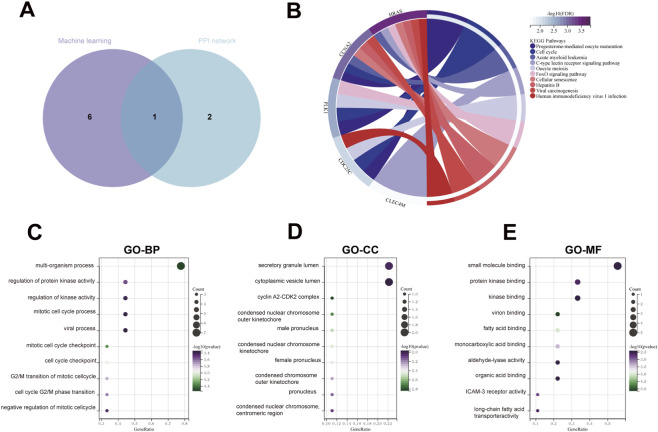
Functional Enrichment Analysis of the Nine Integrated Core Genes. **(A)** The Venn diagram illustrates the overlap of candidate genes identified by PPI network topology analysis and machine-learning models. **(B)** Chord diagram of the top enriched KEGG pathways for the nine core genes. **(C–E)** Bubble chart of the top enriched GO terms.

To elucidate the biological roles of the identified core genes, a comprehensive Gene Ontology (GO) and KEGG pathway enrichment analysis was performed using the nine key genes identified through the integration of PPI network topology and machine learning models (Figure 7A). The KEGG analysis indicated that these genes were mainly enriched in signaling pathways critical to tumorigenesis, such as Progesterone-mediated oocyte maturation, Cell cycle, and Acute myeloid leukemia (Figure 7B). These pathways are closely related to aberrant proliferation and checkpoint dysregulation, suggesting that PET exposure may promote hepatocarcinogenesis through activation of cell-cycle–related cascades.Consistent with these findings, GO enrichment revealed significant associations with multi-organism processes, secretory granule lumen, cytoplasmic vesicle lumen, and small molecule binding (Figures 7C–E).

### 3.7 Immune cell infiltration analysis in core target genes of PET induced HCC

**FIGURE 8:**
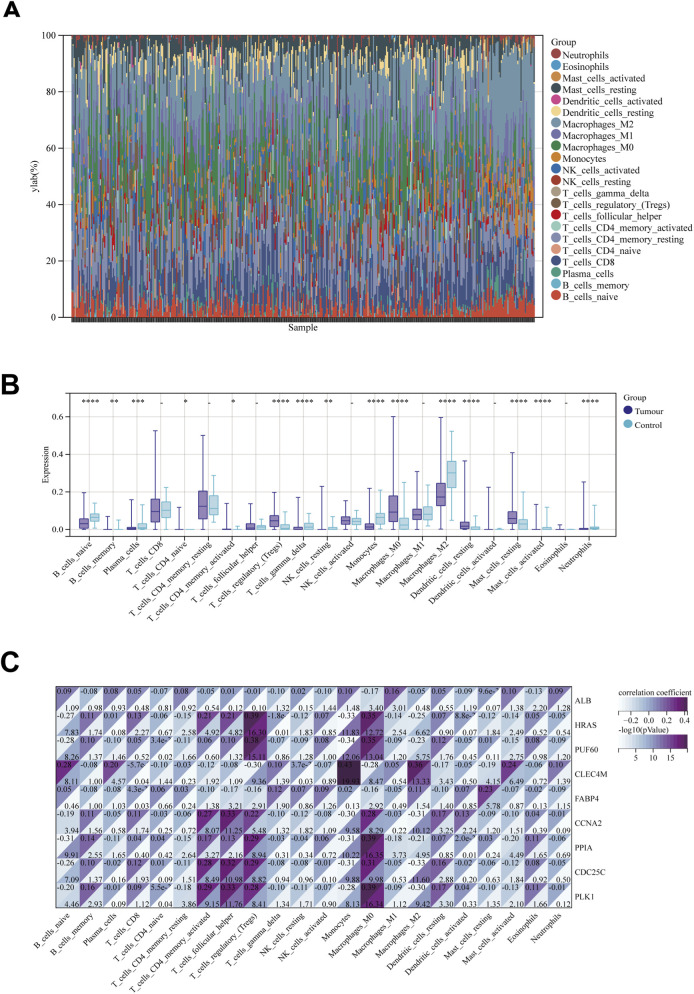
Immune Cell Infiltration Analysis in HCC in the Context of PET-Associated Core Genes. **(A)** Stacked bar plot of 22 immune cell subsets estimated by CIBERSORT. **(B)** Box plots comparing the infiltration levels of immune cells between groups. **(C)** Correlation heatmap between the expression of the nine core genes and immune cell infiltration.

To further elucidate the immunological mechanisms potentially involved in PET-associated hepatocarcinogenesis, we applied CIBERSORT to TCGA HCC RNA-seq data to estimate the relative fractions of 22 immune cell subsets. The inferred infiltration landscape exhibited marked inter-tumor heterogeneity (Figure 8A). Comparative analyses between PET-associated and control HCCs identified statistically significant differences across multiple populations (Figure 8B), including naïve and memory B cells, plasma cells, CD8^+^ T cells, resting and activated memory CD4^+^ T cells, follicular helper T cells, resting NK cells, monocytes, M0/M1/M2 macrophages, resting dendritic cells, resting mast cells, and neutrophils, indicating a broad remodeling of the tumor immune microenvironment. Correlation analyses further linked immune infiltration patterns with the expression of nine core target genes (Figure 8C). PLK1, CDC25C, CCNA2, and PPIA showed positive correlations with neutrophils, monocytes, and/or M0 macrophages, whereas FABP4 and CLEC4M displayed negative correlations with naïve/memory B cells, plasma cells, follicular helper T cells, and resting NK cells. HRAS expression correlated positively with M1 and M2 macrophages, while ALB exhibited inverse correlations with monocytes and M0 macrophages. No statistically significant association was observed for PUF60. Collectively, these findings suggest that the PET-associated core genes are correlated with distinct immune infiltration patterns in HCC. Such associations indicate potential links between the molecular targets of PET and immune remodeling, although direct causal effects of PET exposure on the immune microenvironment cannot be inferred from these data.

### 3.8 Molecular docking validation of PET–core gene interactions

**FIGURE 9:**
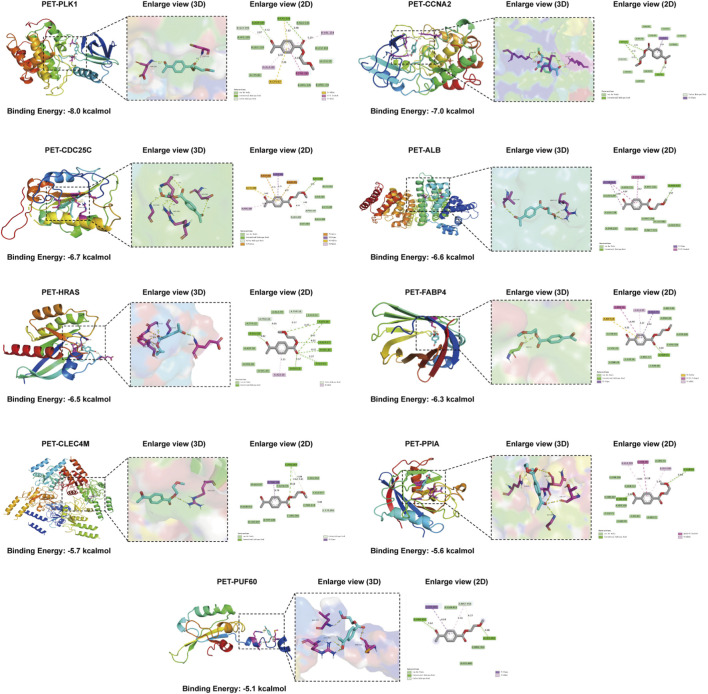
Molecular docking poses and binding free energies between PET and the nine core target proteins.

A comprehensive molecular docking analysis was conducted to investigate the potential binding interactions of PET with the identified core genes. The docking results demonstrated strong and stable binding affinities between PET and all nine target proteins, with binding free energies consistently below −5.0 kcal/mol, indicating spontaneous and energetically favorable interactions. This denotes stable and spontaneous molecular interactions. In molecular docking, binding energies below 0 kcal/mol are indicative of spontaneous binding, and those below −5.0 kcal/mol are considered to represent high-affinity interactions. The stable docking poses of all PET-protein complexes were further confirmed through visualization of their binding conformations (Figure 9). Specifically, PET displayed the strongest binding affinity with PLK1 (−8.0 kcal/mol), followed by CCNA2 (−7.0 kcal/mol), CDC25C (−6.7 kcal/mol), ALB (-6.6 kcal/mol), HRAS (−6.5 kcal/mol), FABP4 (−6.3 kcal/mol), CLEC4M (−5.7 kcal/mol), PPIA (−5.6 kcal/mol), and PUF60 (−5.1 kcal/mol).These interactions provide structural confirmation of the computationally predicted PET–target relationships and substantiate the hypothesis that PET may exert biological effects through direct modulation of these key HCC-associated proteins.

### 3.9 Molecular dynamics and free energy characterization of protein–ligand complexes

**FIGURE 10:**
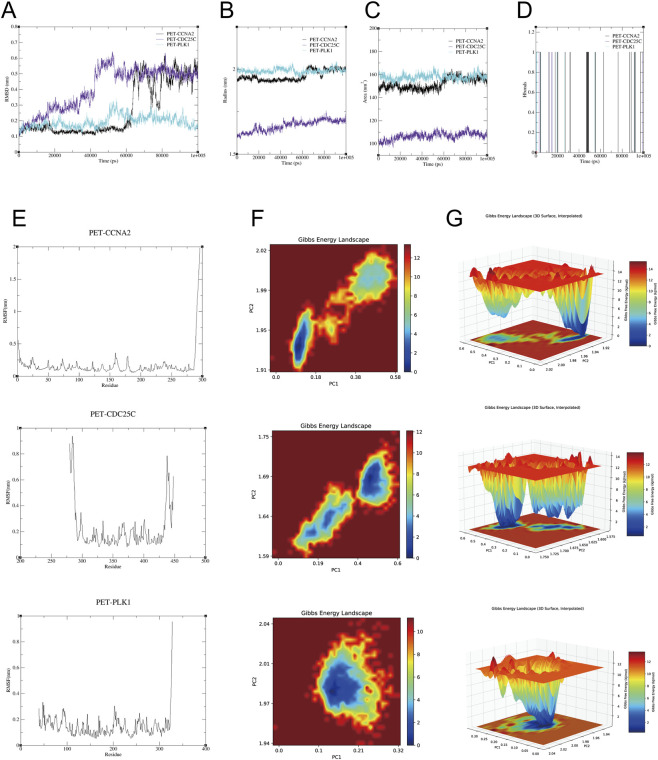
Molecular dynamics analysis of PET–protein complexes. **(A)** RMSD curves of PET-CCNA2, PET-CDC25C, and PET-PLK1 during the 100 ns molecular dynamics simulation. **(B)** Rg changes of the three complexes over the simulation period. **(C)** SASA profiles of the three complexes over time. **(D)** Time-dependent hydrogen-bond counts between each protein and PET. **(E)** RMSF residue fluctuation profiles of the three complexes. **(F)** Two-dimensional Gibbs free-energy landscapes based on PCA. **(G)** Three-dimensional free-energy surfaces of the three complexes.

A 100 ns molecular dynamics simulation was performed for the PET-CCNA2, PET-CDC25C, and PET-PLK1 protein–ligand complexes, and their structural stability, flexibility, and free-energy characteristics were comprehensively analyzed. The RMSD profiles (Figure 10A) show that all three complexes underwent initial structural relaxation and gradually stabilized, with PET-PLK1 displaying the smallest fluctuations and the highest stability, while PET-CCNA2 and PET-CDC25C exhibited minor mid-simulation variations but remained stable overall. The Rg results (Figure 10B) further indicate that PET-PLK1 and PET-CCNA2 maintained relatively constant compactness, whereas PET-CDC25C showed a slight increase, suggesting greater structural flexibility. The SASA values (Figure 10C) reveal that PET-PLK1 and PET-CCNA2 maintained similar and stable solvent-exposed surface areas, while PET-CDC25C exhibited consistently lower SASA values. Hydrogen-bond analysis (Figure 10D) shows intermittent formation patterns across all complexes, reflecting dynamic yet persistent protein–ligand interactions. RMSF analysis (Figure 10E) indicates generally low residue flexibility, with distinct localized fluctuations in each system: PET-CCNA2 exhibited pronounced flexibility at both termini, PET-CDC25C showed peaks around residues 280–300 and 440–460, and PET-PLK1 displayed a single peak near residues 320–330. The two-dimensional free-energy landscapes (Figure 10F) revealed multiple energy basins for PET-CCNA2 and PET-CDC25C, indicating more diverse conformational sampling, whereas PET-PLK1 showed a more concentrated basin. The three-dimensional free-energy surfaces (Figure 10G) were consistent with these findings, showing a single deep energy well for PET-PLK1 and multi-basin features for the other two complexes. Overall, all systems remained structurally stable throughout the simulation, but PET-PLK1 exhibited the greatest conformational stability and energy concentration, whereas PET-CCNA2 and PET-CDC25C showed higher localized flexibility and more diverse conformational states.

### 3.10 Clinical significance of core genes and functional validation via PET exposure

**FIGURE 11:**
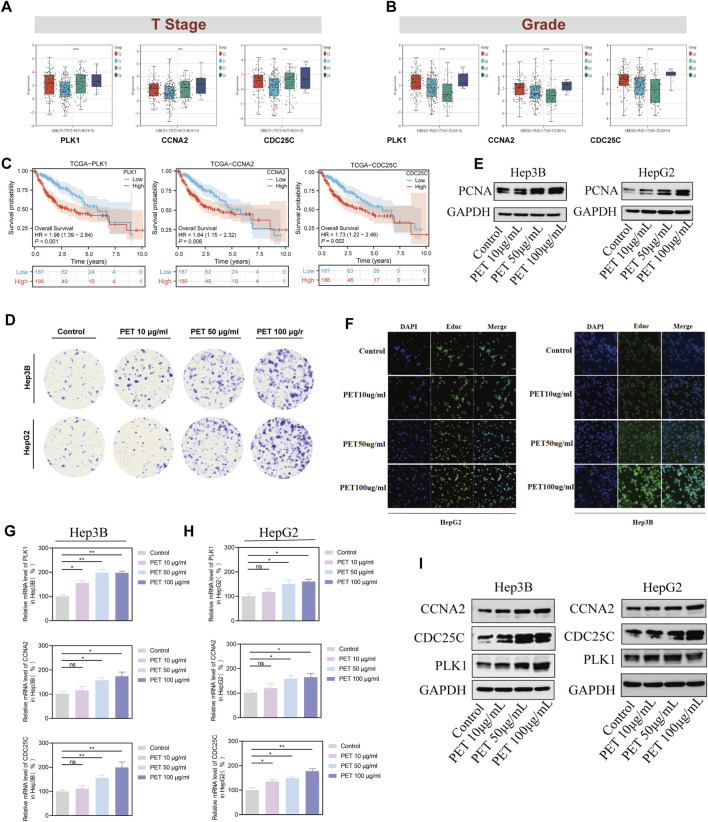
Clinical significance of PLK1, CCNA2, and CDC25C in HCC and functional validation of PET exposure *in vitro*. **(A,B)** Expression of PLK1, CCNA2, and CDC25C in TCGA-LIHC samples stratified by tumor T stage and pathological grade. All three genes showed progressive upregulation with advancing stage and grade. **(C)** Kaplan–Meier survival analysis showing that high expression of PLK1, CCNA2, and CDC25C was significantly associated with poorer overall survival. **(D)** Colony formation assay in Hep3B and HepG2 cells showing that PET treatment enhanced proliferative capacity in a concentration-dependent manner. **(E)** Western blot analysis demonstrating upregulation of PCNA following PET treatment. **(F)** EdU incorporation assay confirming a concentration-dependent increase in DNA synthesis upon PET exposure. **(G,H)** qRT-PCR analysis showing concentration-dependent upregulation of PLK1, CCNA2, and CDC25C mRNA in Hep3B and HepG2 cells. **(I)** Western blot analysis confirming increased protein expression of PLK1, CCNA2, and CDC25C following PET treatment. Data are presented as mean ± SD from three independent experiments. *p < 0.05, **p < 0.01, ***p < 0.001.

In our prior comprehensive analyses, PLK1, CCNA2, and CDC25C were identified as key cell cycle regulatory genes highly associated with PET exposure, demonstrating close links to the occurrence and progression of HCC. To further validate their expression profiles and potential prognostic value in real-world clinical contexts, and to investigate whether PET can induce similar pro-oncogenic phenotypes *in vitro*, we conducted a systematic evaluation combining clinical cohort analysis and functional experiments. Based on TCGA-LIHC clinical data, we first analyzed the expression patterns of these three core genes across different tumor T stages and pathological grades. The results demonstrated that their expression levels progressively increased with advancing tumor stage and higher malignancy; expression was significantly elevated in patients with advanced-stage (T3–T4) or high-grade (G3–G4) tumors compared to those with early-stage (T1–T2) or low-grade (G1–G2) tumors (all P < 0.05) (Figures 11A,B). Further Kaplan–Meier survival analysis revealed that high expression of PLK1, CCNA2, and CDC25C was consistently associated with significantly poorer overall survival (OS) (HR = 1.98, 1.64, and 1.73, respectively; all P < 0.01) (Figure 11C).

To validate whether PET exposure could induce gene activation and functional alterations consistent with the aforementioned clinical observations, we treated Hep3B and HepG2 cells with varying concentrations of PET. Colony formation assays demonstrated that PET exposure significantly enhanced the proliferative capacity of both cell lines in a concentration-dependent manner, with the most pronounced effect observed at 100 μg/mL (Figure 11D). Additionally, we assessed PCNA, a well-established marker of cell proliferation, which was also significantly increased following PET treatment across both cell lines (Figure 11E). To directly assess the impact of PET exposure on cell proliferation, EdU incorporation assays were performed. PET treatment significantly increased the percentage of EdU-positive cells in a concentration-dependent manner in both Hep3B and HepG2 cells, with the most pronounced effect observed at 100 μg/mL (Figure 11F). These results provide direct evidence that PET promotes DNA synthesis and HCC cell proliferation. qRT-PCR analysis confirmed that PET significantly upregulated the mRNA expression of PLK1, CCNA2, and CDC25C in both cell lines, with the most substantial upregulation observed in the high-dose group (Figures 11G,H).To further validate these findings at the protein level, Western blotting was performed on Hep3B and HepG2 cells following PET treatment. Consistent with the mRNA expression data, protein levels of PLK1, CCNA2, and CDC25C were significantly upregulated in both cell lines upon PET exposure (Figure 11I).

## 4 Discussion

This study comprehensively investigated the potential molecular mechanisms linking polyethylene terephthalate (PET) exposure to hepatocellular carcinoma (HCC) development. By integrating compound target prediction, transcriptomic profiling, network pharmacology, machine learning–based screening, and molecular docking validation, we identified a series of key genes and signaling pathways potentially mediating PET-induced hepatocarcinogenesis. The findings suggest that PET may promote HCC progression by perturbing metabolic homeostasis, accelerating cell cycle progression, and reshaping the tumor immune microenvironment.

Our study utilized three complementary computational platforms (ChEMBL, PharmMapper, and SwissTargetPrediction) to predict potential molecular targets of PET, yielding 235 candidate proteins after deduplication. This multi-database integration strategy offers several advantages over single-platform approaches. Each prediction algorithm employs distinct methodologies—ligand-based similarity searching, pharmacophore mapping, and reverse docking—thereby capturing different aspects of potential protein-ligand interactions. The convergence of predictions across multiple platforms substantially reduces the likelihood of false positives while maximizing coverage of biologically relevant targets. This approach has been successfully applied in previous studies investigating environmental toxicants and drug repositioning, demonstrating its validity for identifying disease-relevant molecular targets.

Our analysis consistently identified cell cycle regulatory genes—particularly PLK1, CCNA2, and CDC25C—as core mediators of PET-associated hepatocarcinogenesis across multiple analytical approaches including PPI network topology, machine learning feature selection, and molecular docking validation. PLK1 emerged as the strongest PET-binding target (−8.0 kcal/mol) and demonstrated the highest conformational stability in molecular dynamics simulations. PLK1 is a master regulator of mitotic progression, controlling multiple cell cycle checkpoints including mitotic entry, spindle assembly, and cytokinesis (Kalous and Aleshkina, 2023). Its overexpression has been extensively documented in various malignancies, including HCC, where it correlates with aggressive phenotypes and poor prognosis (Tian et al., 2020). The significant upregulation of PLK1 following PET exposure in our Hep3B cell model, coupled with enhanced colony formation capacity, suggests that PET may directly promote hepatocellular proliferation by aberrantly activating mitotic machinery. CCNA2 functions as an essential regulatory subunit of cyclin-dependent kinases (CDKs), governing both S-phase progression and G2/M transition (Zhang et al., 2019). Our finding that CCNA2 expression increases with tumor grade and stage, and that high expression predicts poor survival, aligns with previous reports establishing cyclins as critical drivers of hepatocarcinogenesis (Wang et al., 2024). The robust binding affinity between PET and CCNA2 (−7.0 kcal/mol) and the observed upregulation following PET treatment suggest that environmental PET exposure may disrupt normal cell cycle checkpoint control, facilitating uncontrolled proliferation. CDC25C is a dual-specificity phosphatase that activates CDK1 by removing inhibitory phosphorylations, thereby triggering mitotic entry (Sur and Agrawal, 2016). Dysregulated CDC25C expression compromises the G2/M checkpoint, allowing cells with genomic damage to proceed through division—a hallmark of malignant transformation (Liu et al., 2019). The PET-induced upregulation of CDC25C observed in our experiments may therefore represent a mechanism by which environmental plastic exposure promotes genomic instability and carcinogenesis. The convergence of these 3 cell cycle regulators in our analyses, combined with KEGG enrichment showing overrepresentation of cell cycle and progesterone-mediated oocyte maturation pathways (which share molecular machinery with mitotic control), provides compelling evidence that cell cycle dysregulation constitutes a primary mechanism of PET-associated hepatocarcinogenesis.

Beyond cell cycle control, our enrichment analyses revealed significant involvement of metabolic pathways, including pyrimidine metabolism, glycine/serine/threonine metabolism, and small molecule metabolic processes. The identification of FABP4 as a core target is particularly intriguing, as it represents a functional link between lipid metabolism and cancer progression. FABP4 is primarily known for its role in adipocyte differentiation and lipid trafficking, but emerging evidence implicates it in cancer cell metabolism and tumor microenvironment crosstalk (Hao et al., 2018a; 2018b; Yang et al., 2023). Its expression in HCC has been associated with tumor-associated macrophage infiltration and metabolic reprogramming toward lipid utilization (Jin et al., 2021). The negative correlation between FABP4 and certain immune cell populations in our analysis suggests that PET exposure may influence hepatic lipid handling in ways that secondarily affect immune surveillance. Additionally, the enrichment of phosphate-containing compound metabolism and phosphorus metabolic processes aligns with the known role of aberrant nucleotide metabolism in cancer. Altered pyrimidine metabolism, in particular, supports the elevated nucleotide demand characteristic of rapidly proliferating tumor cells, potentially explaining the enrichment of this pathway among PET-associated targets.

The CIBERSORT analysis revealed substantial correlations between the expression of PET-associated core genes and immune cell infiltration patterns in HCC. While these associations do not directly demonstrate that PET exposure reshapes the immune microenvironment, they suggest that genes potentially targeted by PET are linked to immune remodeling in HCC. Notably, PLK1, CDC25C, CCNA2, and PPIA showed positive correlations with immunosuppressive cell populations such as neutrophils, monocytes, and M0 macrophages, while FABP4 and CLEC4M exhibited negative correlations with anti-tumor immune cells including naïve B cells, memory B cells, plasma cells, and NK cells.These findings suggest that PET exposure may not only directly affect cancer cell behavior but also reshape the immune landscape to favor tumor progression. The accumulation of myeloid-derived suppressor cells and tumor-associated macrophages, particularly M2-polarized macrophages, is known to create an immunosuppressive microenvironment that facilitates tumor growth, invasion, and metastasis. Conversely, the reduced presence of cytotoxic lymphocytes and NK cells would impair anti-tumor immune surveillance.Tumor-associated macrophages (TAMs), particularly those with M2-like polarization, are well-established promoters of HCC progression through secretion of growth factors, angiogenic mediators, and immunosuppressive cytokines (Cheng et al., 2022; Dallavalasa et al., n.d.). The correlation of HRAS with both M1 and M2 macrophage infiltration indicates complex immune dynamics that warrant further investigation.

The clinical significance of our identified core genes is underscored by their strong associations with tumor progression markers and patient survival outcomes. The progressive increase in PLK1, CCNA2, and CDC25C expression across advancing T stages and pathological grades indicates their involvement throughout HCC development and progression. More importantly, high expression of these genes consistently predicted significantly worse overall survival, with hazard ratios ranging from 1.64 to 1.98, highlighting their potential utility as prognostic biomarkers. These findings have important implications for HCC risk assessment and management. If PET exposure contributes to HCC development through upregulation of these cell cycle regulators, then individuals with high environmental or occupational PET exposure may represent a high-risk population warranting enhanced surveillance. Furthermore, these genes may serve as therapeutic targets; indeed, PLK1 inhibitors are currently under clinical investigation for various cancers, and our findings suggest they may be particularly relevant for environmentally associated HCC.

The integration of molecular docking and dynamics simulations with experimental validation provides a comprehensive view of how PET may exert its biological effects. The consistently favorable binding energies (all < −5.0 kcal/mol) across all nine core targets indicate that PET can potentially interact with multiple proteins involved in diverse cellular processes. While these *in silico* predictions require further biochemical validation, they provide mechanistic plausibility for the observed biological effects. The 100 ns molecular dynamics simulations of the three most relevant complexes (PET-PLK1, PET-CCNA2, PET-CDC25C) revealed differential stability profiles that may reflect distinct modes of action. The exceptional stability of PET-PLK1, characterized by minimal structural fluctuations and concentrated free-energy landscapes, suggests a high-affinity, relatively rigid binding mode that could lead to sustained modulation of PLK1 activity. In contrast, the more flexible binding profiles of PET-CCNA2 and PET-CDC25C, with multiple energy basins and localized conformational flexibility, indicate more dynamic interactions that may involve conformational selection or induced-fit mechanisms.

PLK1 is a master regulator of mitotic entry, spindle assembly, and cytokinesis. Our molecular docking and dynamics simulations demonstrated stable binding between PET and PLK1 (binding energy −8.0 kcal/mol, sustained structural stability over 100 ns), while *in vitro* experiments confirmed that PET exposure significantly upregulates PLK1 expression in Hep3B cells. Given that PLK1 overexpression is known to drive mitotic dysregulation, centrosome amplification, and genomic instability in HCC, these findings support a causal model in which PET directly interacts with PLK1 and enhances its expression, thereby promoting uncontrolled proliferation. CCNA2 governs both S-phase progression and G2/M transition through CDK activation. Our integrated analyses identified CCNA2 as a hub gene across PPI network topology and machine learning screening. PET treatment upregulated CCNA2 expression in a dose-dependent manner, and KEGG enrichment analysis revealed overrepresentation of the “Cell cycle” pathway among PET-associated targets. These findings suggest that PET exposure may accelerate cell cycle progression by activating CCNA2-mediated checkpoint control. CDC25C activates CDK1 to trigger mitotic entry. Our data show that PET exposure upregulates CDC25C expression, which is consistent with compromised G2/M checkpoint function—a known mechanism promoting malignant transformation. The direct binding interaction between PET and CDC25C (binding energy −6.7 kcal/mol) further supports the plausibility of a direct regulatory effect.

Several limitations should be acknowledged. First, our computational predictions, while validated through multiple platforms and molecular docking, require further experimental confirmation through direct binding assays such as surface plasmon resonance or isothermal titration calorimetry. Second, we acknowledge certain limitations in the experimental validation. While we performed EdU incorporation and PCNA detection to assess proliferative activity, direct assessment of cell cycle distribution via flow cytometry would have provided more definitive evidence of G2/M phase dysregulation. Due to temporary equipment unavailability, we were unable to perform flow cytometric analysis in the current study. The EdU and PCNA data, together with upregulation of G2/M-associated regulators (PLK1, CCNA2, CDC25C), provide indirect evidence supporting cell cycle dysregulation. Future studies should include flow cytometry to directly confirm alterations in cell cycle progression. Additionally, while we validated key findings in HepG2 cells, further validation in additional HCC cell lines and primary hepatocytes would strengthen generalizability.Third, while CIBERSORT provides valuable estimates of immune infiltration from bulk RNA-seq data, single-cell approaches would offer higher-resolution insights into cell-type-specific responses. We acknowledge that our docking and molecular dynamics simulations were performed using the PET monomer, which does not fully represent intact polymer particles, microplastics, nanoplastics, or complex environmental mixtures. The binding affinities reported should be interpreted as predictions for the monomeric unit, and future studies should investigate different degradation products and size fractions.

## 5 Conclusion

This study integrated computational target prediction, transcriptomics analysis, machine learning, molecular docking, and experimental validation to elucidate the mechanisms linking PET exposure to HCC. From 235 PET-interacting proteins, 40 overlapped with HCC-associated genes, and PLK1, CCNA2, and CDC25C were identified as core mediators through network topology and machine learning screening. Molecular dynamics simulations confirmed stable PET-target binding, with PLK1 showing the highest affinity (−8.0 kcal/mol). TCGA-LIHC analysis demonstrated that elevated expression of these genes correlated with tumor progression and poor prognosis. Functional enrichment revealed involvement in cell cycle regulation, metabolic reprogramming, and immune remodeling. Importantly, *in vitro* experiments validated that PET dose-dependently upregulated these genes and promoted colony formation in Hep3B cells. In conclusion, our findings establish PLK1, CCNA2, and CDC25C as key molecular nodes through which PET may promote hepatocarcinogenesis, providing potential biomarkers and therapeutic targets for populations with high PET exposure.

## Data Availability

Public datasets: Available in the The Cancer Genome Atlas (TCGA) database, which can be accessed via the Genomic Data Commons (GDC) portal: https://portal.gdc.cancer.gov/. The specific project is “TCGA-LIHC” (Liver Hepatocellular Carcinoma). Molecular docking data: Ligand (PET) from PubChem(CID 18721140); protein structures from UniProt. All code and analyzed data are accessible upon reasonable request by contacting the corresponding author.
