# Supplementary material for: Assessing the toxicological effects of exposure to polyethylene terephthalate on hepatocellular carcinoma: insights from network toxicology, molecular docking, molecular dynamics, and experimental validation
Source: Front Pharmacol. 2026 Apr 22;17:1772751. doi: 10.3389/fphar.2026.1772751 (PMC13143728; doi:10.3389/fphar.2026.1772751)
Supplement: Supplementary file 2 [file Table1.docx]

**Supplementary table 1. The primer sequences in this study**

| Primer name | Forward | Reverse |
| --- | --- | --- |
| CCNA2 | CAGAAAACCATTGGTCCCTC | CACTCACTGGCTTTTCATCTTC |
| PLK1 | TGACTCAACACGCCTCATCC | GCTCGCTCATGTAATTGCGG |
| CDC25C | CTGGCAAGGATTTTCACCAGG | ATGTGCAGATGTGCTACGCT |
| GAPDH | GGTGGTCTCCTCTGACTTCAACA | GTTGCTGTAGCCAAATTCGTTGT |
